# Auxetics and FEA: Modern Materials Driven by Modern Simulation Methods

**DOI:** 10.3390/ma17071506

**Published:** 2024-03-26

**Authors:** Russell Galea Mifsud, Grace Anne Muscat, James N. Grima-Cornish, Krzysztof K. Dudek, Maria A. Cardona, Daphne Attard, Pierre-Sandre Farrugia, Ruben Gatt, Kenneth E. Evans, Joseph N. Grima

**Affiliations:** 1 Metamaterials Unit, Faculty of Science, University of Malta, MSD 2080 Msida, Malta; russell.galea.15@um.edu.mt (R.G.M.); grace.muscat.16@um.edu.mt (G.A.M.); james.grima.16@um.edu.mt (J.N.G.-C.); maria.cardona@um.edu.mt (M.A.C.); daphne.attard@um.edu.mt (D.A.); pierre-sandre.farrugia@um.edu.mt (P.-S.F.); ruben.gatt@um.edu.mt (R.G.); 2 Institute of Physics, University of Zielona Gora, ul. Szafrana 4a, 65-069 Zielona Gora, Poland; k.dudek@if.uz.zgora.pl; 3 Centre for Molecular Medicine and Biobanking, University of Malta, MSD 2080 Msida, Malta; 4 Department of Engineering, Faculty of Environment, Science and Economy, University of Exeter, North Park Road, Exeter EX4 4QF, UK; k.e.evans@exeter.ac.uk; 5 Department of Chemistry, University of Malta, MSD 2080 Msida, Malta

**Keywords:** auxetics, negative Poisson’s ratio, metamaterials, finite elements

## Abstract

Auxetics are materials, metamaterials or structures which expand laterally in at least one cross-sectional plane when uniaxially stretched, that is, have a negative Poisson’s ratio. Over these last decades, these systems have been studied through various methods, including simulations through finite elements analysis (FEA). This simulation tool is playing an increasingly significant role in the study of materials and structures as a result of the availability of more advanced and user-friendly commercially available software and higher computational power at more reachable costs. This review shows how, in the last three decades, FEA proved to be an essential key tool for studying auxetics, their properties, potential uses and applications. It focuses on the use of FEA in recent years for the design and optimisation of auxetic systems, for the simulation of how they behave when subjected to uniaxial stretching or compression, typically with a focus on identifying the deformation mechanism which leads to auxetic behaviour, and/or, for the simulation of their characteristics and behaviour under different circumstances such as impacts.

## 1. Introduction

The last three decades have seen an extensive growth in our knowledge about various new materials and metamaterials, more known as auxetics [1], which exhibit the rather unusual mechanical property of a negative Poisson’s ratio [2,3]. Auxetic materials defy common expectations by expanding laterally when uniaxially stretched, rather than becoming thinner. Conversely, these materials become thinner, rather than thicker, when uniaxially compressed. In fact, the Poisson’s ratio measures the magnitude of this effect [4] and is defined for a particular cross-section of a material in the plane *Ox_i_*-*Ox_j_* in terms of the applied strain *ε_i_* in the direction *Ox_i_* and the transverse strain *ε_j_* in the orthogonal transverse direction *Ox_j_* as

(1) $$\nu_{ij}=-\frac{\varepsilon_{j}}{\varepsilon_{i}}$$

In general, for an isotropic three-dimensional (3D) material, the Poisson’s ratio can assume any positive or negative value. However, for isotropic materials, this Poisson’s ratio is restricted to have values −1 ≤ *ν* ≤ +0.5 (−1 ≤ *ν* ≤ +1 for two-dimensional (2D) orthotropic) [5]. Materials which are auxetic in all planes for loading in any direction have been termed as “complete auxetics” whilst materials which are only auxetic in specific planes for loading in specific directions (common in crystalline materials and cubic metals [6,7]) have been termed “partially auxetic” [8,9,10]. 

Auxetics have associated with them a number of benefits and enhanced properties that are not that commonly encountered in most everyday materials [11]. They are highly desirable for their exceptional properties, including high indentation resistance [12,13,14,15,16,17,18,19,20], high fracture toughness [20,21,22,23], shear resistance [18,24,25], energy absorption [26,27] and other enhanced dynamic characteristics [28,29,30,31]. These unique attributes enable auxetics to find diverse applications across various fields such as in the medical field [32,33,34,35,36,37], sportswear applications [38,39,40,41,42], military defence equipment [43,44,45,46], as well as in the automotive [47,48,49] and aerospace [50,51,52] industries. For example, personal protective equipment (PPE) would benefit from being resistant to indentations. However, traditional materials tend to displace material away from the point of impact, resulting in reduced density upon impact. Auxetics, upon impact, behave in a manner that the material flows towards the point of impact, increasing their density and resisting indentation. 

As a result of these enhanced properties and possible applications, scientists have availed themselves of various research tools to characterize, optimize and design de novo metamaterials which exhibit auxeticity. In this field of research, pure experimental research is not always deemed feasible. This may be attributed, at least in part, to the complex geometries that auxetic structures have, the lack of availability of “off the shelf” auxetic materials on a large scale, issues relating to sustainability, practical drawbacks of physical prototyping (prototypes are expensive, unsustainable, difficult to manufacture, and just a general burden to test) as well as ethical issues which may arise in certain experimental testing in real applications where auxetics could be truly needed (e.g., stents [53,54]). As a consequence, researchers have resorted to rapidly developing research protocols based on computer simulation and numerical analysis, often using experimental results and analytical studies to verify the numerical results obtained [9,55,56,57,58,59,60,61,62,63,64,65,66,67,68,69].

One of the numerical approaches which researchers have used to make significant inroads in the field of auxetics is the finite element method (FEM), a technique that provides an approximate solution indicating how a system behaves when subjected to specific physical constraints. The method provides easy solutions when dealing with problems involving complex geometries and/or nonhomogeneous domains having different properties in different regions. It involves the subdivision of the original domain into smaller parts called elements. The subdomains are easier to treat as they can be chosen so that their governing equations are much simpler than those for the whole domain. These elements are then connected through the nodes that they have in common. Nodes are locations in space. In general, in the case of one-dimensional (1D) elements, they would represent points on a line, while if the elements are 2D or 3D, they represent corners [70]. The shape of the elements is then determined by joining the nodes via straight lines. (Here it should be noted that sometimes elements might also have internal nodes.) Once divided into these subregions, the network of nodes is referred to as a mesh or grid. FEM then involves solving the governing equations locally at the nodes to determine the physical variables at these points. Putting together the local solutions provides a piecewise solution that represents an approximation to the actual one for the whole domain [71]. The accuracy of the solution depends on the size of the elements and increases with the number of nodes used. At the same time, the computational time increases with the number of nodes. Hence, a balance needs to be found between accuracy and processing time. Most frequently, this is established by requiring that the solution that is used does not vary by more than a small percentage (many times taken to be 1%) from that obtained using more nodes. At this point, the solution is considered to be mesh-independent. Once the FEM is applied and the solution obtained, the subsequent analysis is referred to as the finite element analysis (FEA). This method dates back to the 1940s, having been developed to overcome the mathematical difficulties when applying the theory of elasticity [72,73]. In 1956, FEA was adopted by the aerospace industry as it allowed the modelling of complex geometries and provided instant analysis of their mechanical properties [74]. The method provided a solution to many problems of material analysis by calculating the stress within a structure [75]. The FEA method is well-known for its reliability in determining the location, magnitude, and direction of forces, as well as in assigning stresses and deformations. Therefore, within a few decades, the method was adopted by several research fields, ranging from dentistry [76,77,78,79,80] to biomechanics [81,82,83,84] amongst others [85,86,87,88]. 

A major advantage of using FEA as an integral part of a research protocol to study auxetics during the design stages is that it reduces the need for prototypes, by providing a quick, non-invasive and repeatable analysis [89,90,91]. Moreover, apart from the obvious sustainability advantages FEA brings with it when used for the optimization of material design whilst reducing experimental waste, FEA offers the advantage that it is an excellent tool to simulate and animate the “deformation mechanism” of auxetics, thereby providing researchers with essential information on how the deformations are leading to auxetic behaviour. This also applies to more complex systems such as 3D negative Poisson’s ratio mechanical metamaterials, that are not only usually elastically stable under large compressive deformations but are also capable of exhibiting diverse properties by changing the feature size, making them potential candidates for both functional and structural applications [92]. This statement was equally relevant before the advent of 3D printing, as it is now when the current expectations regarding the performance capabilities of materials, metamaterials and structures have grown tremendously. Moreover, FEA offers the advantage that it is not limited to the conventional test parameters and can accurately and reliably simulate real-life scenarios even with unconventional parameters, such as an isotropic negative Poisson’s ratio. This is particularly useful in the field of auxetics to study the behaviour of these unconventional materials in practical applications (e.g., shearing [93] or pressing [13]). Despite the lack of real materials with such properties, FEA enables us to anticipate their behaviour. Also, FEA can be used to investigate scenarios which would be difficult to achieve physically. One such study examined re-entrant hexagonal honeycombs and their post-yield behaviour under tension. Through FEA, the study revealed a plastic collapse mechanism and identified three stages of force–displacement curves [64]. 

**Figure 1:**
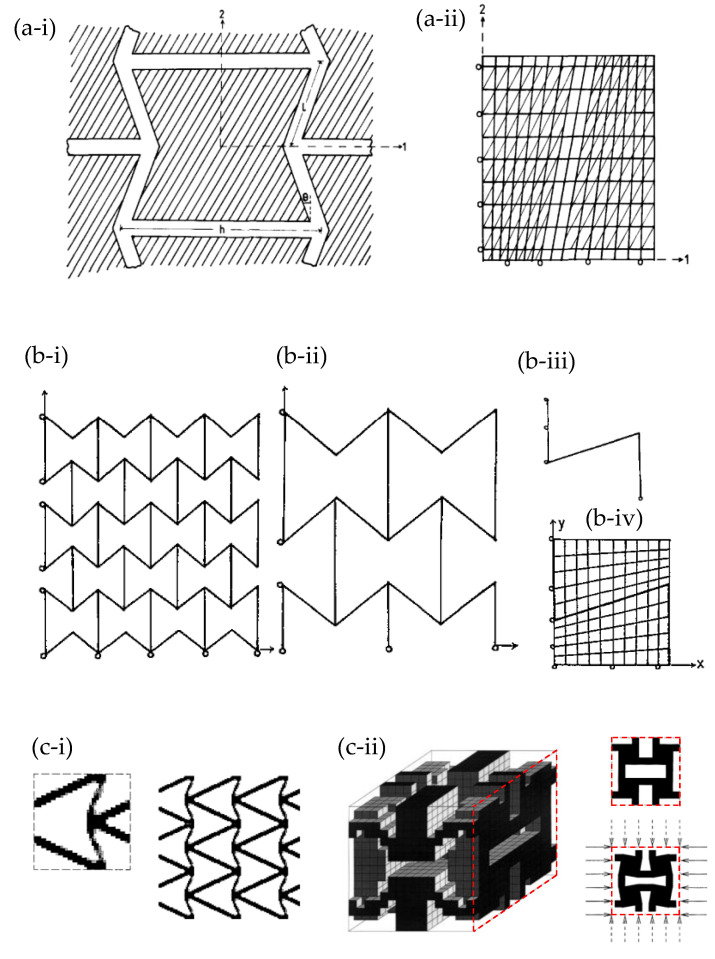
Some of the earliest uses of FEA to model auxetics: (**a**) Evans et al.’s (1992) matrix embedded re-entrant network-embedded fibre composite [94] with (**a-i**) showing the unit cell and (**a-ii**) showing a finite element grid used a typical re-entrant network composite with fibre re-enforcement being unshaded and matrix shaded; (**b**) various FEA representations of the analogues for the molecular level systems [95] where (**b-i**,**b-ii**,**b-iii**) correspond to systems with a different number of representative repeat units constructed from beam elements whilst (**b-iv**) represents a system where the honeycomb is embedded within an ultra-soft matrix having a near zero Young’s modulus; (**c**) examples of the auxetics motifs generated by topology optimization by Sigmund, where (**c-i**) shows the double-V auxetic motif [99] and (**c-ii**) shows the motif which is now referred to as the anti-tetrachiral motif [101].

FEA has been utilised in some of the earliest seminal papers on negative Poisson’s ratio by Evans and his group who used the software ANSYS (Ansys Inc., Houston, TX, USA) to model two-dimensional re-entrant honeycombs as an embedded fibre in composites [94] (Figure 1a) and as a template for molecular level systems [95] (Figure 1b) to model 3D auxetic foams [96], to study continuous fibre-reinforced composites where either reinforcing or matrix constituents could have a negative Poisson’s ratio [97] and to model auxetic microporous polymers [98]. Ole Sigmund [99] used a topology optimization protocol and a method based on a finite-element discretization of the base cells to propose and analyse various classical auxetic motifs. These included the double-V re-entrant system [100] and another motif [101], later referred to as the “anti-tetrachiral” motif [102,103,104] (Figure 1c). Nowadays, much more developed commercially available software such as ANSYS (Ansys Inc., Houston, TX, USA), MSC Marc (Hexagon AB, Newport Beach, CA, USA) and ABAQUS (Dassault Systèmes Simulia Corp., Johnston, RI, USA) combined with high computational power are essential tools when conducting research in the mechanical properties of materials and has, thus, been utilized by several research groups, as noted in Appendix A, in the study of auxetic metamaterials [63,105,106,107,108,109,110,111,112,113]. It is also most useful that various textbooks have been written to guide researchers through the background theory and application of FEA [70,71,114].

Recognizing the growth and development of auxetics, concurrently with finite element analysis, this review examines some of the major discoveries in the field of auxetics which have been made through FEA, and shows how this method of analysis contributes to the shaping of our current knowledge about negative Poisson’s ratio. 

## 2. Simulation of Auxetic Structures

The diverse range of enhanced properties associated with auxetics, together with their potential for use in several practical applications meant that, in the last four decades, considerable effort has been made to design and develop novel or improved auxetic structures. In fact, as clearly stated by [115], there is a constant “demand for new types of auxetic structures to achieve different design goals of their use (dynamic behaviour, fatigue resistance, manufacturability)”. 

If one had to look at the modus operandi which has been used to generate and optimise auxetics, one can clearly identify various approaches, including what may be referred to as the “systematic geometry-based approach” and the “topology optimisation approach”. The former approach may be explained through the specific example of the 2D re-entrant geometry motif shown in Figure 1a, which has long been recognised for its potential as an auxetic system [1,116,117]. The systematic approach typically starts with the identification of geometric parameters which characterise the system, in this case, the length of the slanting ligament (l), the length of the ligaments connecting them (h) and the angle between them (θ). It then examines how each of these parameters, and/or combinations of them, affects Poisson’s ratio. This can be achieved through, for example, the formulation of analytical models based on assumptions of how the systems deform (e.g., flexure, hinging and/or stretching of the ligaments) and/or running a series of FEA simulations where each parameter is varied whilst keeping the other parameters fixed. Both the analytical and simulation approaches offer distinct advantages and disadvantages. Analytical models lead to mathematical expressions for Poisson’s ratio in terms of geometric parameters. On the other hand, the systematic FEA approach allows for a more realistic representation of systems, as discussed elsewhere [118]. The topology optimisation approach, first applied and pioneered in the field of auxetics in the 1990s by Sigmund and co-workers [100,119,120], has the capability of automatically achieving an optimal structure and material layout (geometry) with a negative Poisson’s ratio subject to specific pre-defined constraints through the use of advanced algorithms. [115,121] Such constraints include the desired macroscopic properties and prescribed performance. For example, this approach can help identify optimal structural configurations that maximize the desired properties of auxetic materials, such as impact resistance or energy absorption, while maintaining or reducing material weight. Typically, the optimised structures obtained have their properties evaluated through FEA before potentially being manufactured as prototypes for experimental testing [122,123]. Various studies have applied the technique within the field of auxetics, including a study by Clausen et al. [124] who employed parameterized optimization to produce auxetic chiral topologies which maintain their desired Poisson’s ratio over strains; a study by Bruggi et al. [125] who made use of SIMP-based topology optimization (i.e., solid isotropic material with penalization) to obtain auxetics based on micropolar materials; and a study by Wang et al. [126] who applied isogeometric topology optimization with the aim of reducing stress concentrations within star-shaped auxetic structures. 

### 2.1. Two Dimensional Systems

A substantial number of studies on auxeticity were devoted to investigating the mechanical properties of two-dimensional motifs that are capable of exhibiting a negative Poisson’s ratio. This focus on 2D systems is probably due to the fact that the Poisson’s ratio *ν_ij_* can essentially be regarded as a 2D property. 

The re-entrant honeycomb geometry, shown in Figure 1, is one of the most studied auxetic motifs. This structure essentially represents a standard hexagonal honeycomb where the classical Y-shaped joints have been inverted to form arrow-shaped joints. Such systems are known to exhibit a negative Poisson’s ratio when loaded on-axis, primarily due to flexure (or hinging) of the slanting ligaments. This motif, equally useful as a 2D system and as a model for particular cross-sections of 3D materials, was one of the earliest auxetic systems studied by FEA around three decades ago [94,95,96] (Figure 1a,b). Since then, numerous modified re-entrant structures were tested through FEA to diversify and/or optimise the geometry [127,128,129] and to analyse the resulting mechanical properties such as energy absorption capacity [127], synclastic behaviour [130,131] and impact resistance [132,133,134].

**Figure 2:**
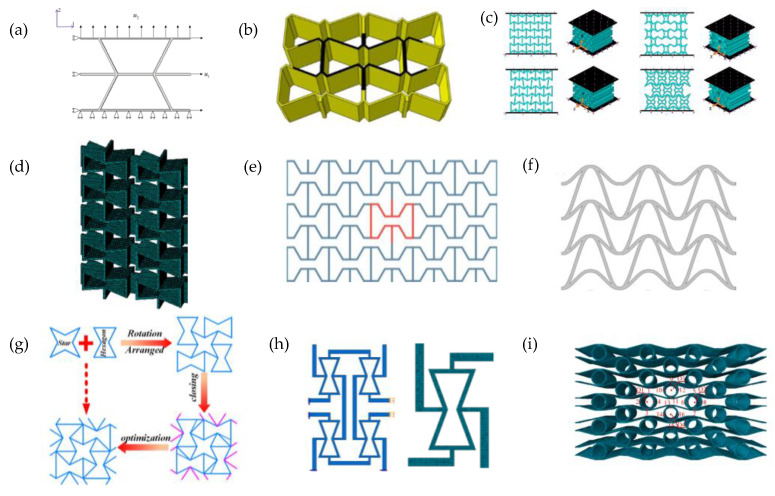
Examples of modified re-entrant structures, which can be seen as variations in the units in Figure 1, proposed by (**a**) Lu et al. (2016) [140]; (**b**) Bezazi et al. (2005) [135] and Harkati et al. (2017) [128]; (**c**) Gohar et al. (2021) [127]; (**d**) Huang et al. (2017) [141]; (**e**) Khan et al. (2019) [59] and Mustahsan et al. (2022) [142]; (**f**) Guo et al. (2020) [143]; (**g**) Wang et al. (2023) [144]; and (**h**) Zhang et al. (2021) [145] and (**i**) Li et al. (2022) [129].

Numerous other studies have utilised FEA to investigate several variations based on the 2D re-entrant hexagonal honeycomb systems and Sigmund’s double-V model (Figure 1c-i) to simulate their mechanical properties, including in-plane Poisson’s ratio and Young’s modulus under uniaxial loading [135,136,137,138,139]. Through FEA simulations, several studies were carried out on the shape optimization of the re-entrant honeycombs by analysing the effect of a number of variables on the physical properties. For instance, Lu et al. in 2016 [140] analysed a re-entrant honeycomb with an additional narrow rib in the unit cell, resulting in a cellular structure with significantly enhanced Young’s modulus [140]. Bezazi et al. in 2005 [135], and the subsequent work by Harkati et al. in 2017 [128], investigated the system illustrated in Figure 2b. Most notably, Bezazi et al. reported that for certain internal angles, the proposed structure exhibits a decrease in the Poisson’s ratio when compared to the conventional honeycomb. Also, the presence of edge corners in the proposed configuration gives rise to a cellular structure with enhanced flexibility compared to the classical centrosymmetric one [135]. In the follow-up work, Harkati et al. [128] investigated the shear and axial deformation using FEA, providing a better insight into the deformation mechanism for the auxetic behaviour and the geometric parameters governing them, specifically the cell wall thickness [128]. Moreover, Gohar et al. [127] used FEA to optimize and model various novel auxetic structures under compressive loading (Figure 2c), systems which they then constructed using 3D printing and tested physically. These included a set of novel re-entrant structures, termed mixed-star structures. These re-entrant structures exhibited a number of superior properties, including a high energy absorption capacity [127]. 

Similarly, Huang et al. [141] investigated a new type of honeycomb design consisting of two distinct parts, a re-entrant hexagonal component, and a thin plate section, illustrated in Figure 2d. The authors developed theoretical models describing the in-plane uniaxial tensile modulus, shear modulus and Poisson’s ratios, and verified them through FEA [141]. FEA was also instrumental in validating analytical and experimental methods, as demonstrated in the work by Khan et al. [59] and Mustahsan et al. [142] (Figure 2e). These used FEA in conjunction with experimental testing to validate the analytical model accounting for the bending, shearing and axial deformation of modified re-entrant honeycomb structures and showed a close agreement between the three methods (numerical, analytical and experimental). Other notable studies include the investigations by Guo et al. (2020) [143] on the double-U honeycomb structure shown in Figure 2f, in which the arrow-shaped system in Figure 1c-i proposed in the 1990s by Ole Sigmund [99] and Larsen et al. [100] was modified to have better load resistance and a higher energy absorbing capacity [143], and the WSH honeycombs composed of stars and hexagons studied by Wang et al. (2023) (Figure 2g) which exhibited excellent energy absorption capacity and enhanced anti-impact behaviour [144]. 

Another study carried out by Zhang et al. investigated two new hybrid metamaterial concepts combining a core unit cell of re-entrant or cross-chiral shape with lateral missing ribs (Figure 2h). FEA simulations optimised specific effective properties, while non-linear simulations were used to study the Poisson’s ratio and stiffness of these metamaterials under large deformations [145]. Similarly, Li et al. proposed the composite auxetic structure consisting of corrugated sheets and tubes, shown in Figure 2i. This was studied using FEA, in conjunction with experiments and theoretical analysis, to investigate the variables affecting the Poisson’s ratio and the mechanical properties of the structure, as well as shape optimization [129]. Other rather complex 2D systems studied include the hierarchical re-entrant honeycombs by Zhan et al. (2022), who used FEA to show that these systems exhibit enhanced mechanical properties under compression. Here, analysis of the deformation shows that when the system is compressed, the addition of a second order triangular hierarchy converts the deformation mechanism from bending-dominated to stretching-dominated, and revealed a combination of deformation mechanisms which contributed to significant improvement in the mechanical properties [146]. 

**Figure 3:**
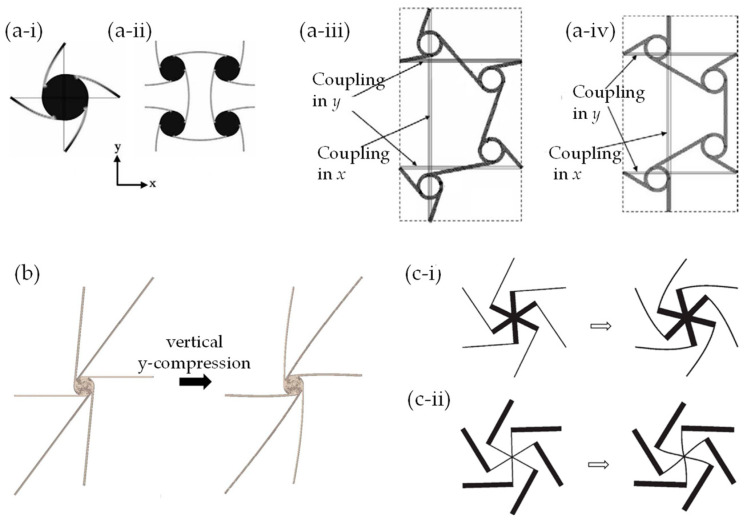
Examples of auxetics with chiral building blocks or based on rotating rigid units obtained via slits or perforations: (**a**) the repeat units in the regular (**a-i**) tetrachiral, (**a-ii**) anti-tetrachiral (**a-iii**) trichiral and (**a-iv**) anti-trichiral, as presented in the work by Alderson et al. (2010) [104]; (**b**) the repeat unit and the manner it deforms as predicted by FEA of an irregular hexachiral [159]; (**c**) FEA-simulated deformations of uniaxial compression in the vertical y-direction of (**c-i**) hexachirals, where the star-shaped node is much more rigid than the ligaments, and (**c-ii**) starchirals, where the ligaments are much more rigid than the nodes with the consequence that deformations occur within the nodes (the “starchiral mechanism”) [150].

The FEA method has been employed to explore various other auxetic mechanisms including chiral systems and rotating rigid units. In particular, inspired by work the of Lakes [147,148] and Sigmund et al. [101], FEA was a key tool in studying 2D periodic systems characterised by highly ordered chiral sub-units [102,103,104,129,149,150,151,152,153,154,155,156,157] (such as the ones in Figure 3a,c), as well as systems with disordered or irregular chirals [158,159] (such as the system in Figure 3c). 

Here, FEA was used to carry out parametric studies to optimise the geometries of the proposed chiral systems and to investigate their mechanical properties such as auxeticity, energy absorption capacity, shear resistance and much more. The unifying design aspect of these chiral systems is that they generally consist of rigid nodes to which thin flexible ligaments are attached in a manner that they form a chiral building block. As discussed by Alderson et al. [104], to model this system through a representative volume unit, it is essential to apply the appropriate boundary conditions and constraints (see Figure 3a-iii,a-iv as an illustration of typical constraints applied). Furthermore, as highlighted by Mizzi et al. [160], the correct application of the boundary conditions often dictates the success or otherwise of a simulation. Simulations of systems with a chiral building block such as the ones shown in Figure 3a–c demonstrate that auxeticity from the “chiral mechanism” occurs when the ligaments are flexible and the nodes are rigid. Generally, when the system is subjected to uniaxial compression, the nodes with the ligaments attached to it rotate, thus, constraining the ligaments to flex in synchrony (to some extent or another). This synchronized mode of deformation causes a lateral contraction which in turn results in auxeticity, something which is even evident in the irregular hexachirals simulated by FEA by Mizzi et al. (2018). [159] However, when the nodes are much less rigid than the ligaments, deformations occur predominantly in the nodes, as demonstrated by Attard et al. (see Figure 3c-ii) [150]. The authors referred to this mechanism as the “starchirals mechanism”.

**Figure 4:**
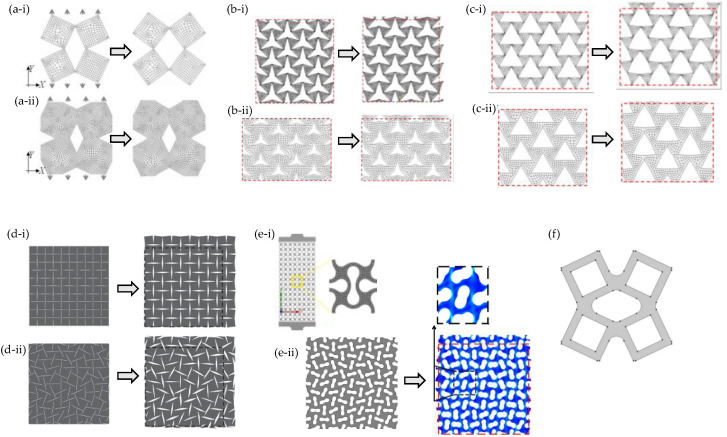
Examples of perforated systems which mimic rotating rigid units: (**a**) systems with diamond-shaped perforations where system in (**a-i**) has minimal overlap compared to system in (**a-ii**); (**b**) systems with star-shaped perforations where system in (**b-i**) has minimal overlap compared to system in (**b-ii**); (**c**) systems with triangle-shaped perforations, where system in (**c-i**) has minimal overlap compared to system in (**c-ii**); (**d**) systems with slits (regular (**d-i**) and randomly oriented (**d-ii**)); (**e**) system with (**e-i**) regularly and (**e-ii**) irregularly placed peanut-shaped perforations; and (**f**) implementation of a rotating squares model as proposed by Mrozek and Strek et al. (2022) [173]. Systems in (**a**,**d**,**e**,**f**) mimic the rotating squares model, whilst (**b**,**c**) mimic the rotating triangles model. Note that, if applicable, systems are being loaded vertically.

FEA was also one of the main tools used to explore how slits and perforations could generate a negative Poisson’s ratio [161,162,163,164,165,166,167]. Notably, early studies on this idea (Figure 4a–c) highlight how strategically placed perforations could transform a regular sheet to an auxetic or zero-Poison’s ratio system by making it mimic the “rotating squares” [168] and “rotating triangles” [169] auxetic mechanisms. This could be achieved when using appropriately placed diamond-shaped (Figure 4a) [161], slit [162] (Figure 4d), star-shaped [164] (Figure 4b) or triangular-shaped [164] (Figure 4c) perforations. Other similar studies investigated the mechanical properties and deformation mechanisms of “rotating rigid units”-mimicking systems. Amongst others, Wang et al. (2021) [170] and Atilla Yolcu et al. (2022) [171] explored such systems designed through the use of regularly and irregularly peanut-shaped perforations (depicted in Figure 4e); Acuna et al. [172] simulated rectangular perforations; whilst Mrozek and Strek [173] (Figure 4f) investigated a system of perforations mimicking the rotating squares model [64,174,175,176]. Afshar et al. utilised FEA to investigate non-porous perforated rotating rigid units having a soft inclusion in the perforations. It was shown that these inclusions still retained a negative Poisson’s ratio but reduced the extent of auxeticity. This would be useful in applications of non-porous auxetic materials [105]. Similar conclusions were also drawn in an earlier FEA study by Mizzi et al. (2015) [177] which examined non-porous grooved single-material systems. 

### 2.2. Three Dimensional Systems

3D auxetic materials and models, ranging from crystalline materials such as zeolites, silicates, etc. [178,179,180] to macroscopic systems such as the Hoberman sphere [181] are gaining importance due to their abundance, versatility, properties and potential applications. However, their added complexity compared to their 2D counterparts, can make the interpretation of their Poisson’s ratio more challenging. In 3D auxetics, the overall deformations might result from multiple auxetic mechanisms acting simultaneously, as evident in some of the earliest seminal work in the field (Figure 1c-ii) [101]. FEA, thus, becomes an even more indispensable tool to elucidate their intricate behaviour. In fact, FEA has been extensively used to investigate a wide range of 3D auxetics including cellular materials [129,171,182], composites [183,184,185,186,187] and some rather novel constructs such as a surface auxetic structure (SAS) [188]. 

**Figure 5:**
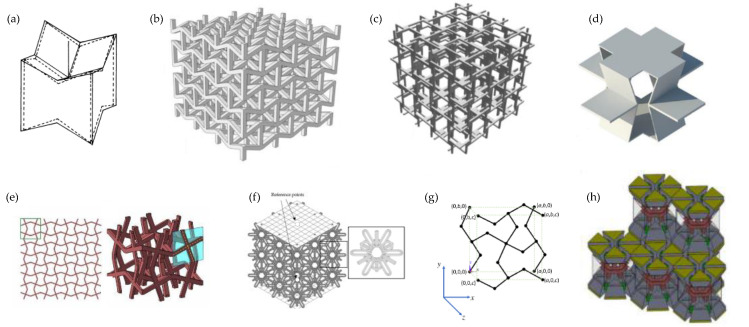
Examples of 3D cellular structures studied via FEA in the last three decades: (**a**) work by Evans, Nkansah and Hutchinson, 1994, on a cellular systems to simulate the microstructure of auxetic foams [96]; (**b**) work by Wang et al., 2017, on a re-entrant version of the classical re-entrant honeycomb [191]; (**c**) another 3D version of the re-entrant honeycomb as studied by Nasim and Etemadi, 2018 [192]; (**d**) the 3D chiral cellular structure as proposed and studied by Farrugia, Gatt and Grima, 2019 [193]; (**e**) a 3D cellular system as studied by Wang et al., 2020, which can be seen as a 3D render on the “rotating squares” [194]; (**f**) a 3D cellular structure proposed, modelled and tested experimentally by Photiou et al., 2021 [196]; (**g**) a mechanical version of a crystalline system modelled by Grima Cornish et al., 2022 [197]; and (**h**) a sliding system investigated by Su et. al. (2021) [199].

FEA has offered diverse insights into the effects of different geometric parameters on the mechanical properties and deformation mechanisms in 3D systems from as early as the 1990s. The diversity of systems studied can be appreciated from some cellular systems shown in Figure 5. Evans et al. [96] used the elongated dodecahedron as a model for auxetic foams (Figure 5a). More recently, Yang et al. [189] and Wang et. al. [190,191] studied a 3D re-entrant honeycomb (Figure 5b); Nasim and Etemadi [192] proposed a cellular structure (Figure 5c); Farrugia et al. (2019) [193] explored a novel 3D anti-tetrachiral honeycomb Figure 5d; and Wang et al. (2020) [194] investigated “3D cross-chiral auxetic materials”, a system where some of its 2D projections bear a very close resemblance to the “rotating squares” [168] profile with the squares replaced by crosses [195] (Figure 5e). Later, in 2021, Photiou et al. worked on the so-called “tetra-petal auxetic [196] (Figure 5f), while in 2022, Grima-Cornish et al. [197] examined the crystalline material framework of boron arsenate as if it were a purely mechanical system (Figure 5g). Recently, it has also been proposed that 3D auxetic systems may be constructed by introducing continuous voids having constant cross-sectional areas at particular loci in different planes [198]. Collectively, these works highlight how FEA simulations have become the staple tool to bridge design and mathematical modelling with experimental work and physical testing. Moreover, for a given idealised design or concept, FEA has made it possible to obtain a glimpse into how real systems might behave, thereby guiding experimentalists towards the synthesis of systems with tailor-made auxetic properties. All this contributed significantly to the growth of the field of auxetics over the last decades. While most 3D cellular systems exhibit their auxetic behaviour through “re-entrant” or “rotating rigid units” (or its chiral variant) mechanisms, Su et. al. investigated a 3D-printable auxetic metamaterial operating through a sliding mechanism (Figure 5h). Their experimental and FEA simulations produced coherent results, and the proposed structure was claimed to exhibit superior performance to the 3D re-entrant honeycomb, due to higher compression resistance and more stable auxetic behaviour [199]. 

**Figure 6:**
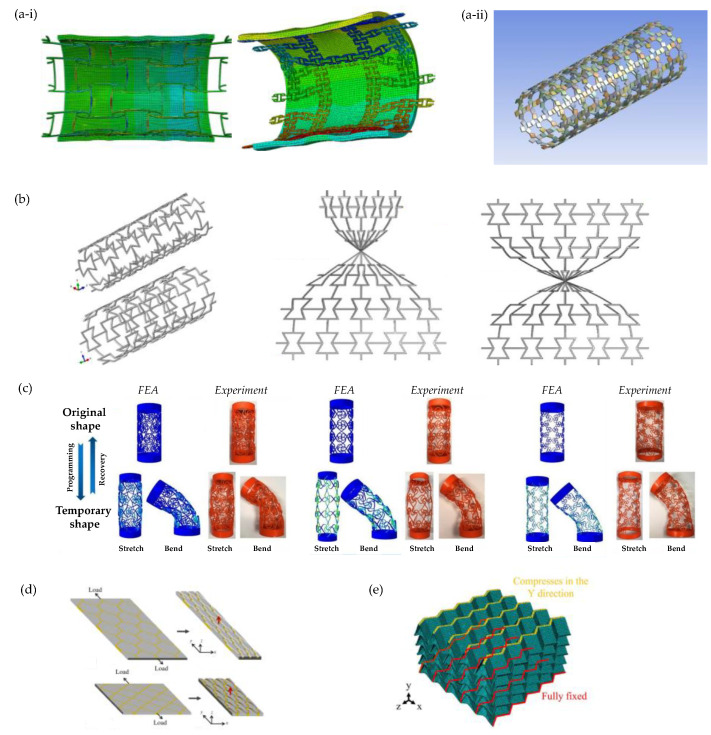
Other examples of 3D auxetics studied via FEA: (**a**) simulations by Wu et al. [62] of the mechanical properties of (**a-i**) regular and hierarchical anti-tetrachiral auxetic stents, the latter being based on the design concept by Gatt et al. [200] shown in (**a-ii**); (**b**) 2022 work by Changfang et al. [188] on surface auxetic structures; (**c**) 2022 work by Wan et al. [201] on programmable auxetic metamaterials with shape memory effects; (**d**) honeycomb composites with auxetic out-of-plane characteristics as proposed and modelled by Grima et al. [183]; and (**e**) 2023 work by Li et al. [187] on the auxetic and failure characteristics of composite stacked origami cellular materials under compression.

From a design and modelling perspective, the possibility to make use of the third dimension brings with it some very interesting possibilities on how to achieve auxeticity. FEA facilitates the transition from concept to an actual physical model. Thus, tubular structures were made by morphing novel or existing 2D auxetics into the shape of a tube for potential use as biomedical devices like artery stents. Wu et al. (2018) [62] (Figure 6a-i) followed the design principles proposed by Gatt et al. [200] (Figure 6a-ii) to explore the mechanical properties of the proposed artery stents. Understanding such properties is crucial for the mechanical integrity and biomechanical performance reliability of the stent–plaque–artery system. Wu et al. proposed two innovative chiral stent types with auxetic properties: an anti-tetrachiral stent with circular and elliptical nodes, and a hierarchical anti-tetrachiral stent with circular and elliptical nodes (Figure 6a). FEA was employed to study the effects of stent geometrical parameters on the tensile mechanical behaviour of the proposed stents. It was deduced that the proposed anti-tetrachiral stent can be tailored by adjusting the levels of hierarchical structures and unit cell design parameters. FEA was used to study the deformation mechanism during stenting. The proposed structures exhibited remarkable radial expanding abilities while maintaining axial stability, which is ideal for such applications [62].

Other studies of this type include work on the surface auxetic structures (SAS) studied by Changfang et al. (2022) [188] (Figure 6b). These structures are obtained by morphing 2D auxetic surfaces into 3D shells. FEA was employed to study two types of SAS: RAS (reversed auxetic structure) and CAS (crimped auxetic structure). The focus was on simulating the compressive behaviour of both plane and surface auxetic structures to understand their mechanical behaviour and energy absorption characteristics. Through this study, it was deduced that RAS displayed the auxetic effect of compression shrinkage as well as the super-mechanical effect of compression twist. Such behaviour was found to appear only in the local positions of the beams, giving the structure great potential in engineering applications [188]. Recent work by Wan et al. delved into four-dimensional (4D) printed programmable auxetic metamaterials with shape memory effects, using both FEA (see Figure 6c) and experimental approaches [201]. The analysis revealed that the proposed cylindrical shells possess desired mechanical properties and configurations, indicating potential applications as biomedical scaffolds [201]. 

FEA has also proven particularly valuable when analysing auxetic composite structures. This is evident from the early contributions to the field by Evans et al. [94,97]. Grima and co-workers [183] used FEA simulations to demonstrate how an auxetic 3D composite consisting of metal honeycombs embedded in a soft rubbery matrix could exhibit a negative Poisson’s ratio out-of-plane by forcing the softer matrix to move out of the pores whilst the honeycomb is stretched (Figure 6a). Through FEA, researchers could investigate the effects of changes in framework geometry in relation to changes in the Poisson’s ratio both in-plane and out-of-plane. This enabled the optimisation of composite parameters for maximum auxetic behaviour [183]. Additionally, these simulations led to an analytical study outlining the requirements for auxetic behaviour. 

Recent work on composites also includes that of Li et. al. (2023) [187] who used FEA to investigate the mechanical and auxetic characteristics of a composite fibre-reinforced stacked origami structure (Figure 6d). It was deduced that composite stacked origami structures have lower density and better energy absorption characteristics compared to those made from metal using additive manufacturing processing [187]. Another interesting work is that on cementitious composites [184], where FEA-based machine learning was used to generate accurate predictions of the auxetic behaviour in cementitious composites [184]. 

## 3. Simulation of Responses on Auxetics under Dynamic, Quasi-Static Loading, Impact and Indent Loading

The use of FEA as a tool to simulate the responses of auxetic materials and structures has been pivotal, primarily due to its efficiency and time/cost-effectiveness compared to actual physical testing as it reduces the need for production and testing of experimental prototypes [192,202]. FEA’s efficiency lies in its ability to accurately acquire the desired mechanical responses and to analyse the behaviour of different sections of the material in a manner which is sometimes difficult to perform experimentally in a non-destructive manner. This is crucial when assessing the response of the material to applied loads and other desirable properties as required by the intended application. 

The properties of auxetics are inherently linked to their geometry (including topology) and deformation mechanism (which may be controlled through a number of ways, such as materials contrast [203]). To achieve the desired properties, researchers often investigate the effect of the geometric parameters on the mechanical properties and behaviour of auxetics under specific conditions. Recently, there has been growing interest in the mechanical properties and energy absorption capacity of auxetic materials under dynamic and quasi-static loading. One of the key challenges in this area is to develop a deeper understanding of the factors that influence these properties under different loading conditions and the deformation modes of specific auxetic structures, challenges which may be successfully addressed using FEA. 

**Figure 7:**
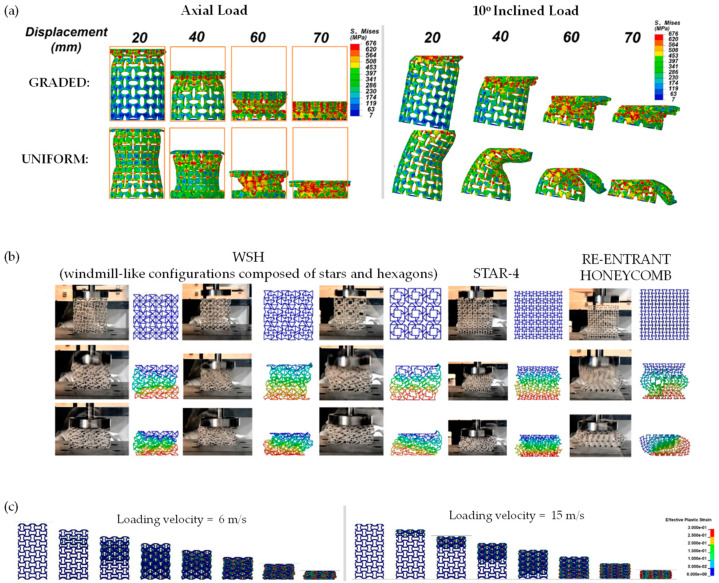
Examples of dynamic simulations: (**a**) deformation modes of a gradient auxetic tube and uniform auxetic tube under axial and inclined loads for 3D auxetics studied via FEA by Han et al.’s 2022 work [204]; (**b**) work by Wang et al. (2023) showing a comparison of experimental and FEA deformation diagrams under dynamic impact behaviour with an impact velocity of 1.7 m/s [144]; and (**c**) uniaxial compression at different velocities of the regular chiral as simulated by Novak et al. [207].

In a 2022 study, Han et al. [204] used FEA to investigate the mechanical properties and deformation modes of gradient and uniform auxetic tubes subjected to axial and inclined loads (Figure 7a). Their findings revealed that the gradient auxetic tube had better energy absorption capacity and higher strength compared to the uniform auxetic tube [204]. Similarly, in the same year, Han, Ren et al. used FEA to investigate a design for ribbed metamaterials with high-quality energy-absorption capability speeds [205]. In a more recent study, Wang et al. [144] conducted numerical simulations to study the static and dynamic plateau stresses of windmill-like (WSH) honeycombs and their energy absorption capacity (Figure 7b). Comparing these with the STAR-4 auxetic honeycomb and the standard re-entrant honeycombs, they found that the windmill-like (WSH) honeycombs had excellent energy absorption performance under both static and dynamic loading conditions [144]. In the study by Chen et al., a set of auxetic lattices with enhanced stiffness were proposed. This was achieved by adding a strengthening rib into conventional auxetic unit cells in a direction perpendicular to the re-entrant direction. The effective mechanical properties of these variants were calculated using the fast Fourier transform-based homogenization method, which illustrated that their Young’s modulus in 2D can be improved by approximately 200% along the strengthening direction without significantly sacrificing the auxetic characteristics. However, such an enhancement is weakened in 3D. The paper provides insight into the design of the new structures of unit cells with enhanced stiffness and a negative Poisson’s ratio [206]. 

Novak et al. [18,207] explored the mechanical behaviour of chiral auxetic [207] (see Figure 7c for the results of the simulation of a regular chiral auxetic under dynamic uniaxial compression at different velocities) and graded/non-graded [18] cellular structures under different loading conditions. Their investigation included quasi-static low-velocity dynamic compression and high-strain rate loading and shearing scenarios. The study employed experimental measurements, infrared thermography, high-speed camera images and computational simulations to examine the deformation mechanism of chiral auxetic structures. Computational simulations were used to obtain a more detailed analysis of mechanical behaviour at different strain rates and estimations of plateau stress at arbitrary loading velocities. As a result of this analysis, Novak et al.’s work provides insights into the use of chiral auxetic structures in crashworthiness, ballistics and blast protection applications [18,207].

Other noteworthy studies that have made use of dynamic and quasi-static compression, as well as different impact velocities, include several modified re-entrant diamond structures which exhibited a superior specific energy absorption [208,209]. Similarly, investigations of modified re-entrant honeycombs exhibited potential for crashworthiness applications [210,211]. Additionally, studies examining the energy absorption capacity of star-circle honeycomb structures led to the development of different design strategies for the auxetic honeycomb [212]. 

**Figure 8:**
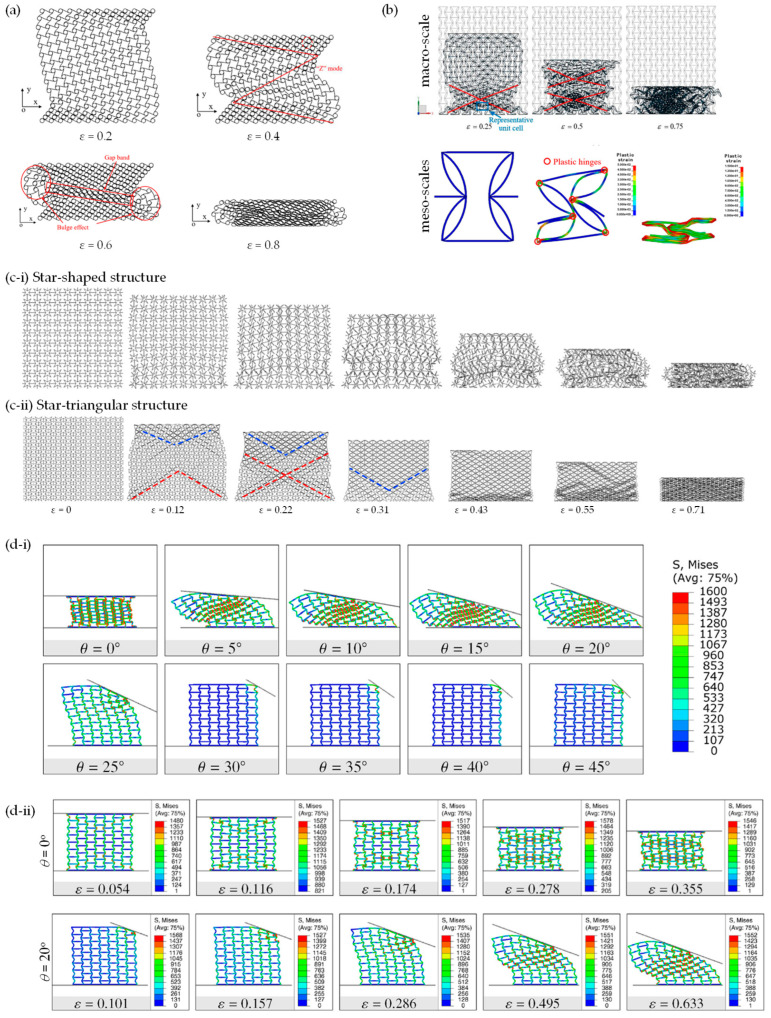
The crushing of auxetics as simulated by FEA: (**a**) quasi-static crushing at different extents of the global strains of tetrachiral honeycomb as summated by Qi et al. [154] at a velocity of 1 m/s; (**b**) predicted deformation processes of a re-entrant circular auxetic honeycombs specimen at typical states in the macro- and mesoscale as simulated by Qi et al. [214]; (**c**) the crushing of “star” honeycombs as simulated by Wei et al. [215]; (**d**) static inclined compression of re-entrant honeycomb auxetic structure as simulated by Singh et al. [216], where (**d-i**) shows the effect of angle of the inclination *θ* of how the load is applied and (**d-ii**) focuses on the system where *θ* = 0°, 20°, showing the deformation at different extents of applied strain.

FEA has also been implemented to expose such materials to dynamic and static crushing conditions. Li et al. conducted a study to analyse the in-plane uniaxial and biaxial crushing characteristics of three honeycombs through explicit dynamic FEA. This research aimed to compare the deformation mode, plateau stress, energy absorption and impact response [213]. In a 2019 study by Qi et al. [154], the in-plane crushing response of tetrachiral honeycombs was investigated under both quasi-static and dynamic loading conditions. This revealed the different modes of deformation in response to the different loading conditions [154] (Figure 8a). A subsequent study by the same authors [214] (Figure 8b) made use of the FEA-predicted deformation to reveal the underlying mechanisms by analysing patterns in the internal stresses for a re-entrant honeycomb with petal-shaped inclined ribs, which they termed as “re-entrant circular auxetic honeycombs”. The article identifies three distinctive regions in their unit cell configuration map, each corresponding to a mesoscale interaction pattern and a macro-scale deformation mode [214]. 

Other studies analysing the crushing performance through FEA include a paper published in 2020 by Wei et al. [215] investigating the deformation upon the crushing of star-shaped honeycombs and a new type of auxetic honeycomb “star” structure (termed star-triangular honeycombs) (Figure 8c). Another study by Singh et al. [216] used FEA to investigate the deformation mechanisms observed during the static inclined compression of the re-entrant honeycomb auxetic structure (Figure 8d), a mode of loading which is likely to have a number of practical applications. This research introduced a novel method to extract micro deformation mechanisms under inclined loading, which were related to the macro deformation regime and the overall mechanical response of the re-entrant honeycomb structure. Through the identification of elements undergoing the plastic strain of more than 10%, micro modes were successfully identified [216]. Incline loading on re-entrant honeycomb systems has also been studied in work by Dhari et al. [217]. Furthermore, a 3D re-entrant structure was also analysed under dynamic crushing conditions to observe its behaviour under extreme deformation [218]. 

Auxetic materials are also associated with impact resistance and have been proposed in applications of protective gear and automotive bumpers. Over the recent years, FEA has seen extensive use in exploring how auxetic materials behave in impact scenarios, significantly aiding material testing across industries such as automotive and aerospace industries. FEA offers a repeatable, non-destructive and rapid test, in lieu or in conjunction with the more time-consuming experimental tests. FEA protocols commonly investigate how auxetic materials respond to impact by analysing the dispersal and redirection of the force, as well as the energy absorption properties. This has proven particularly useful in analysing the crushing patterns and the deformations. For instance, Liu et al. showcased the behaviour of re-entrant auxetic honeycombs under different loading speeds [219]. Meanwhile, Guo et al. examined the impact of velocity and indenter size on a double U honeycomb structure (Figure 2f), and found it to exhibit superior energy absorption capacity and stress distribution compared to the conventional counterpart [143]. Similar to the work above, FEA has been instrumental in assessing the impact velocity and crashworthiness of auxetic structures, including a number of honeycombs [220] and modified honeycombs [144], hierarchical honeycombs [211] and chiral auxetic structures [152]. 

A number of studies have used FEA to investigate the effect of indentation on the deformation mechanism [17]. In recent studies, FEA served to validate findings on the indentation resistance of the hexagonal honeycombs [221] and the indentation behaviour of 3D-printed auxetic reinforced composites [222]. Moreover, a recent study by Attard et al. looked at indentation from the perspective of the indenter. FEA was employed to examine the response of a finger-like indenter made from an inner hardcore (representing bone) surrounded by a softer outer layer (representing flesh) when pressing on a hard sample lined with an auxetic or conventional softer materials. This study revealed that although the auxetic material, with a highly negative Poisson’s ratio, may feel harder when compared to a conventional material of the same Young’s modulus, the auxetic does not have the “bottoming up” disadvantage and, thus, a thinner layer of auxetic can replace a much thicker conventional protective layer. Another emerging direction of studies [223] is the examination of the dynamic properties of auxetics. This is due to a multitude of potential applications that include acoustic absorbers [224,225,226,227], seismic insulators [228,229,230,231], energy harvesting devices [232,233] and artificial intelligence [234,235]. To assess the dynamic properties of the system through the FEM simulations, the first step typically corresponds to the determination of its phononic band structure. A common approach in this case is the use of a single unit cell with Floquet periodic boundary conditions. The resulting phononic band structure provides a plethora of information about the properties of the system ranging from the group and phase velocities of waves propagating through the system [236] to the phononic band gaps [237,238,239], as well as other more complex topological effects [240,241,242]. The possibility of finding the phononic band gaps is of practical importance since it allows the determination of ranges of frequencies at which waves are not transmitted through the system. The Bragg band gaps [243] can be observed once the effective wavelength of the wave propagating through the structure is approximately twice as large as the lattice constant of a metamaterial acting as a phononic crystal [244,245,246]. Over the years, a multitude of studies have been conducted on this topic to identify different classes of metamaterials that could act as wave insulators at various ranges of frequencies. These studies have been reported for devices at very different size scales [247] that corresponded to considerable band gaps at a broad range of frequencies including megahertz [248], kilohertz [249,250,251,252] as well as even lower frequencies related, for example, to seismic applications [228,253,254].

An important aspect that merges the worlds of static and dynamic properties of metamaterials is the possibility of controlling the phononic band structure through structural reconfiguration. In the case of a majority of metamaterials, to significantly modify the phononic band structure and the corresponding band gaps. it is necessary to change the system either by modifying the dimensions of its structural components [255] or by artificially changing the mass of the system, for example, by adding elements having a nonzero mass [256,257]. Despite the effectiveness of these approaches, these methods share the limitation that it is impossible to change the behaviour of the system without fabricating it again. There are also literature examples of metamaterials where the quasi-static mechanical reconfiguration influences the system to the point that its vibration modes change compared to the initial structure. For such scenarios, the entire band structure can indeed be considerably modified. It should be noted that this goal, to a moderate extent, can be achieved even for relatively simple geometries that are often studied from the perspective of their static properties [258,259,260,261]. Nonetheless, a greater change in the band structure can typically be observed for more complex or multistable systems that can be controlled even via external stimuli [262,263,264]. 

In conclusion, the studies reviewed here demonstrate the usefulness of FEA in analysing the mechanical properties and energy absorption capacity of these materials. FEA was pivotal in illustrating the great potential that auxetics have for applications requiring high-energy absorption capacity, such as in the automotive and aerospace industries. 

## 4. Design of Products

FEA excels at replicating testing scenarios and analysing the behaviour of material within a specific scenario. Its application is not limited to replicating what is typically measured in conventional lab testing; rather, it can be extended to replicate the conditions in which the product is expected to perform, enabling the observation of its response. 

Chow et al. proposed the use of 3D-printed thermoplastic polyurethane (TPU) with an auxetic architecture insert for pressure therapy to treat hypertrophic scars. The auxetic structure was designed to easily accommodate the contours of the human body during joint movements. The concept was initially tested in FEA examining the synclastic effect of out-of-plane bending. Subsequently, the formability, structural deformation and auxetic response of re-entrant and double arrowhead auxetic structures were numerically evaluated, followed by an experimental prototype. The study successfully demonstrated how the design was able to facilitate a stable level of pressure during body motion, promoting the recovery of hypertrophic scars [265]. 

**Figure 9:**
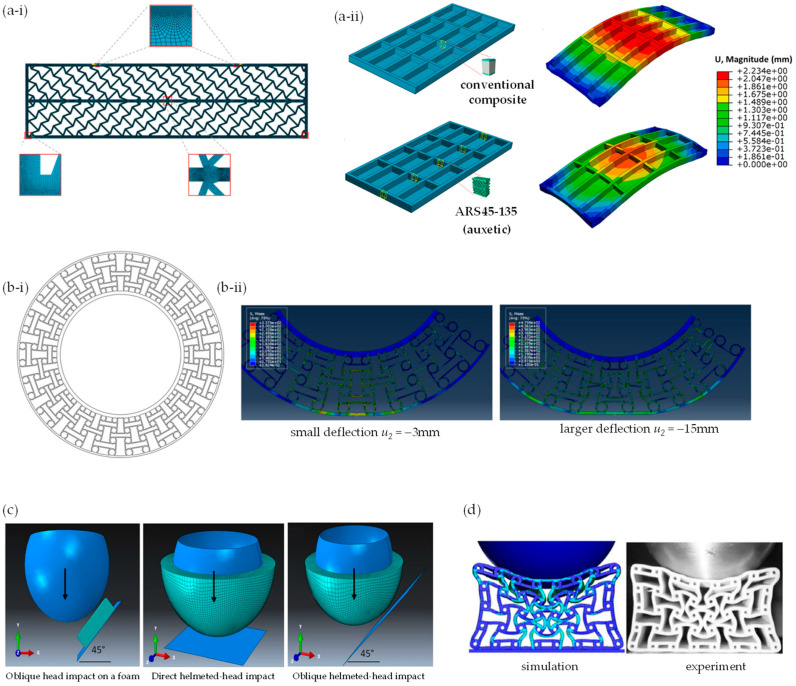
FEA of auxetics and product design: (**a**) auxetic beams for use in the construction industry by Menon et al. (2022) [266], where (**a-i**) shows the ARS45-135 auxetic beam and (**a-ii**) compares this beam to a conventional sandwich-panel beam in lightweight bridge construction; (**b**) non-pneumatic tyres investigated by Wu et al. (2022) [274], where (**b-i**) shows the profile in 2D and (**b-ii**) shows the results of FEA-simulated deformations; (**c**) helmet design as studied by Mosleh et al. (2018) [278] who examined direct and oblique head/helmet impacts; and (**d**) a comparison of FEA with simulated impact for application in sports equipment as reported by Shepherd et al., 2020.

In the construction industry, Menon et al. used FEA to observe the deflection behaviour of basic auxetic re-entrant beams and proposed an improvement to auxetic beam designs, comparing them with traditional beams when used in constructing a lightweight bridge (Figure 9a). The FEA analysis showed that the new auxetic beam designs exhibited better properties with minimal deflection, enhanced load-bearing capacity and a 64% reduction in material [266]. Another study [267] also used FEA to investigate auxetic honeycomb sandwich panels for structural applications. These panels offered reduced weight and displayed a remarkable reduction in the radiated sound power level due to the sandwich structure with an auxetic core [267]. It is important to note that FEA simulations of auxetics extend beyond mechanical scenarios such as uniaxial loading, shearing, pressing, crushing or indenting to encompass more complex scenarios essential for advanced applications. In particular, FEA was extensively used in acoustic and vibration frequency analysis of auxetics. For instance, in two studies by Li et al., the authors developed and applied a FEM to evaluate the propagation of acoustic and elastic waves through 3D phononic crystals. The method accurately computed band structures and identified band gaps and eigenmodes. The results showed that the finite element method was precise and efficient for computing band structures of complex phononic crystal structures with irregular unit shapes and could provide accurate results with commercial finite element code [268,269]. The research has been subsequently used by numerous studies [270,271] for numerical and experimental investigations of phononic crystal structures and the design of novel acoustic devices. 

In a study by Li et al., fundamental frequencies were modelled for a structure comprising sandwich plates with a functionally graded (FG) auxetic 3D lattice core. Non-linear FEA revealed that the effects of FG configurations and strut incline angles are significant, with the FG-X specimen exhibiting the highest fundamental frequency. The study also investigated the large amplitude vibration characteristics of sandwich plates with an auxetic FG 3D lattice core in different thermal environments. Using full-scale non-linear FEA simulations, the effects of FG configurations on the natural frequencies, non-linear-to-linear frequency ratios of sandwich plates, and EPR amplitude curves were studied. Results indicate that the FG configurations distinctly affect the linear and non-linear vibration behaviour of sandwich plates, with EPR-amplitude curves stabilising when the vibration amplitude is sufficiently large. Overall, the study sheds light on the vibration behaviour of functionally graded auxetic 3D lattice metamaterials and sandwich plates with such core, offering insights for further investigations [272].

FEA has been applied in several studies to replicate the loading conditions of passenger vehicles to investigate different structures of auxetic non-pneumatic tyres [273,274,275] (Figure 9b). Additionally, it played a role in the design of anti-tetrachiral stents and hierarchical anti-tetrachiral stents with circular and elliptical nodes. Through FEA, the effects of stent geometrical parameters on the tensile mechanical behaviour of these stents were studied [62], as discussed previously.

Given the excellent ability of FEA to mimic high impacts, with adjustable impact velocities, indents and more, FEM has also been used to facilitate and explore the use of auxetics in military and sports equipment. FEA has already been used to predict material and product behaviour under certain conditions and to analyse design parameters in snowboard wrist protectors [276], helmets [41,277,278] and other sports equipment [279,280,281]. Mosleh et al. [278] used FEA to compare three scenarios involving helmets and head impact, namely an oblique head impact on foam at an angle of 45°, a linear impact of the helmeted head at an angle of 0° and a 45° oblique impact of the helmeted head (Figure 9c). In 2020, Airoldi et al. [282] studied foam-filled energy absorbers with auxetic behaviour for localized impacts and compared the results of FEA simulation to experimental results. A more recent study by Chen et al. (2023) employed FEA to investigate the effect of re-entrant arrowhead liners on helmet protection performance [283]. Through this study, it was illustrated that the auxetic lattice liners offer resistance to indentation, thereby enhancing the protection performance of the helmet [283]. 

When developing PPE, it is also crucially important to consider how materials behave when bent. Auxetic materials possess the remarkable ability to adopt a dome-shaped curvature under bending, making them particularly desirable for integration into PPE. Research conducted by Easey et al. employed FEA to explore the dome shape configurations exhibited by various cellular geometries, including re-entrant, arrowhead, tri-chiral and hexagonal patterns [284]. Their study highlighted that auxetic cellular domes demonstrate lower indentation resistance under compressive loading compared to solid counterparts, underscoring their potential suitability for PPE applications [284]. 

Another property sought after in the development of PPE is energy absorption and crashworthiness. Auxetic materials demonstrate enhanced energy absorption and crashworthiness due to their distinctive negative Poisson’s ratio, resulting in compression-induced compaction. This property is also sought after in the automotive and aircraft industries. In a recent study by Tan et al., FEA was employed to study the energy absorption and crushing performance of hierarchical honeycombs [285]. It was illustrated that the electric vehicle crashworthiness is remarkably improved by the application of the auxetic hierarchical crash box [285].

Within this context, FEA has been employed to investigate the effects of unit-cell geometry and Poisson’s ratio on mechanical properties in auxetic structures and plates. Additionally, FEA has been utilised to analyse the potential of auxetic constituents in composite materials, as demonstrated in the comparison between FEA and experimental analysis (Figure 9d) [286,287]. 

Several studies have investigated the ballistic impact behaviour of auxetic materials. In a study by Novak, chiral auxetic cellular structures were tested to analyse the effect of ballistic velocity and the deformation behaviour of composite sandwich panels. The experimental results validated the computational model of cover plates, which was further utilised to develop computational models of auxetic composite sandwich panels. The study shows that by using the auxetic sandwich panel, the ballistic performance is enhanced compared to monolithic cover plates [288,289]. In a recent study, the ballistic impact behaviour of auxetic sandwich composite human body armour was analysed using FEA. Numerical simulations showed improved indentation resistance and higher energy absorption in the auxetic armour compared to conventional monolithic armour [61]. Similar auxetic sandwich panels were also considered for blast protection in military vehicles, showcasing superior performance both in terms of being more lightweight and offering better protection compared to the solid plate [290]. Additionally, other studies also investigated the use of auxetics in body protection pads [291]. 

## 5. Conclusions, Additional Considerations and Future Directions

This work demonstrated how FEA gradually became an essential tool in developing and understanding the mechanical properties of 2D and 3D auxetic cellular solids, such as honeycomb structures, as well as other auxetic materials, metamaterials and structures. This was only possible through advances in computational sciences. More specifically, FEA is particularly valuable in understanding auxetic behaviour, enabling the precise identification of stresses and strains within materials and metamaterials when loaded, which may not be easily demonstrated through experiments [292]. It is also especially beneficial to simulate characteristics which can only be tested using expensive equipment. 

With time, FEA was used to simulate more real and complex scenarios required for end-product design. Some key studies attempted to optimise the geometry of auxetics to elicit superior properties. Thus, FEA was used to optimise the shape of novel auxetic structures and to determine their mechanical properties prior to prototyping and experimental testing. Since the early years of auxetics, FEA emerged as a fundamental research tool for quick and reliable analysis during the design stage, reducing the need for physical prototyping, thereby accelerating advances in the field. Further advances are still needed, particularly in niche scenarios such as the exploration of new material compositions, granular materials [293], or hybrid auxetic structures combining different mechanisms of auxeticity. Such investigations could further optimize the existing properties of auxetic materials and even uncover new potential applications. Moreover, while FEA provides valuable insights into the mechanical behaviour of auxetic materials, it would be beneficial to extend the analysis to more specific and realistic application scenarios. This includes exploring variable environmental conditions, such as temperature or pressure variations, as well as exposure to UV, moisture, chemicals, biological species (e.g., mould), etc. to assess the reliability and durability of auxetic materials under real operational conditions. 

Like any other experimental or simulation method, FEA has its strengths and limitations, and properly appreciating these limitations ensures that any results obtained are not artefacts of the simulation. One of its limitations is its relative complexity. When considering model building and inputting, the accuracy of the simulation results depend on how the system is entered into the FEA package. Structures are modelled as a continuum and, therefore, it is not possible to accurately model molecular systems. Even for substructures which can be treated as a continuum (for example, in metamaterials and high resolution additively manufactured cellular structures), the structures can still be so small that the number of elements and processing capacities outstrip “normal” computing capabilities. Furthermore, to further validate the results of FEA simulations, it is recommended to integrate the work with a greater amount of experimental data. This could involve advanced mechanical testing to verify the response of auxetic materials under various loads and in different environmental conditions. Integrating experimental data could help to better calibrate FEA simulations and increase their accuracy. The validation of such FEM solutions requires good sets of experimental data, but in this area, modelling and simulation are ahead of the experimental base, and experiments might not always be possible/feasible. This is particularly the case where non-linear, non-elastic, time-dependent and other dynamic phenomena (for example, snap-through processes in folded structures) are being modelled. Some other examples of complex systems which may be trivial to represent within an FEA environment include highly parametrised models, which study the effect of geometric parameters on properties (as is normally the case with modelling of auxetics); disordered systems such as cellular foams (auxetic and non-auxetic), where a representative unit does not exist and assumptions/simplifications need to be made; molecular-level systems where, as discussed elsewhere [197], scalability issues become prominent as the molecular-level interactions cannot be adequately represented through a mechanical model; and realistic systems having some degree of imperfections due to the manufacturing process. To address some of these limitations, it is necessary to construct more complex models than what is currently in the literature, something which will increase the computational cost of the simulations. 

Another limitation of FEA is that the quality of the simulations typically relies on the properties of the intrinsic materials. In some cases, these are not easy to define and the material properties are unmeasured (as is the case with the simulations of systems made from hypothetical auxetic materials, e.g., [13,93]) or are entered in an over simplified way, such as by assuming that the material is behaving linearly, thereby mitigating against high computational costs. Such assumptions may reduce the applicability and accuracy of the simulated characteristics, which calls for further validation. 

Two critical aspects in FEA are the boundary conditions applied (which in the case of the modelling of auxetics, includes situations where one needs to apply full periodic boundary conditions) and the manner in which loads/deformations are applied (uniaxial stretching of auxetics is typically simulated by applying a fixed displacement on a boundary to mimic the application of uniaxial strain, or by applying a force to mimic the application of stress). Moreover, successful finite element simulation requires many key factors to be taken into consideration such as mesh size, mesh type and the constitutive relationship of the matrix material. To ensure that these key aspects of the methodology are correctly applied, it is essential to validate the protocol used, for example, by carrying out convergence testing to ensure that the meshing is sufficiently fine, particularly in regions close to points of impact, joints, regions of high-stress concentration, etc., yet not excessively fine to avoid increasing computational requirements unnecessarily.

Future directions for the modelling of auxetic materials are likely to focus on several key areas. There is a great need to better integrate atomic length-scale modelling and microstructural modelling to bridge the gaps between the length scales and time scales. While commercial FEA packages offer sophisticated pre- and post-processing capabilities, molecular-level modelling remains challenging. There is also significant potential for improvement in the modelling of non-linear, non-elastic, large strain, time-dependent and other dynamic phenomena. A major challenge in this area is the lack of sufficiently large sets of experimental data to validate such models. Establishing a free-to-use central database could greatly facilitate progress in this regard. Current FEM also lacks significant “predictive” capabilities, particularly in the development of radically new architectures. It is anticipated that the integration of AI techniques combined with FEM approaches could offer promising avenues for progress in this area. Finally, the field of metamaterials is enabling the development of a much broader range of “anomalous” properties such as a negative Poisson’s ratio, negative thermal expansion coefficients, negative stiffness, negative mass, etc. These novel metamaterials have the potential to transcend traditional thermodynamic constraints. They can interact both mechanically and electromagnetically and have the ability to draw on external energy sources to drive their anomalous behaviour. This lack of constraint effectively bypasses normal physical constraints at the “material” level (although not at the “system” level). FEM and related methods will need considerable development to cope with the complexities of these new scenarios.

On a final note, it is crucial to consider the environmental impact and sustainability in the design and the application of auxetic materials. This could involve conducting a life cycle analysis of auxetic materials, from production, to use, to disposal. Such analysis can promote the development of materials that are not only technologically advanced but also environmentally sustainable. 

## Data Availability

Not applicable.

## Appendix A

**Table A1:** Examples of works on auxetics classified according to the software used.

Program Used	2D/3D	References
ABAQUS	2D	[17,55,58,64,65,111,112,138,139,140,142,143,155,184,185,209,216,219,220,222,266,269]
	3D	[56,60,62,63,66,92,107,109,110,113,127,128,129,134,137,144,145,146,149,151,152,187,188,190,191,192,194,196,199,201,208,210,213,218,272,274,282,290,292]
ANSYS	2D	[94,95,97,135,150,157,158,159,161,162,163,164,165,166,172,174,182,212,221]
	3D	[57,61,96,105,108,131,136,141,160,167,171,177,183,193,267,273]
Other	2F	[59,130,170,173,176,186,211,214,215,270]
	3D	[18,132,133,153,154,189,202,207,265,268,275,276,288,289]

**Table A2:** Examples of works on auxetics classified according to the characteristic studied.

Property Studied	2D/3D	References
Auxeticity and stiffness	2D	[58,64,65,94,95,97,111,130,135,138,139,140,142,143,150,155,157,158,159,161,162,163,164,165,170,172,173,174,176,182,184,185,266]
	3D	[56,57,60,62,63,66,92,108,110,113,128,129,131,134,136,137,141,145,149,160,171,177,183,187,189,190,191,192,193,194,196,199,201,274,275,292]
Energy absorption and indentation, impact and blast resistance	2D	[17,112,209,211,212,214,215,219,220,221,222]
	3D	[18,57,61,63,92,107,109,127,132,133,144,151,152,153,154,188,194,202,204,205,207,208,210,213,218,273,276,282,288,289,290]
Other	2D	[166,269,270]
	3D	[265,267,268,272]
